# Methylome and transcriptome signature of bronchoalveolar cells from multiple sclerosis patients in relation to smoking

**DOI:** 10.1177/1352458520943768

**Published:** 2020-07-30

**Authors:** Mikael V Ringh, Michael Hagemann-Jensen, Maria Needhamsen, Susanna Kullberg, Jan Wahlström, Johan Grunewald, Boel Brynedal, Maja Jagodic, Tomas J Ekström, Johan Öckinger, Lara Kular

**Affiliations:** Department of Clinical Neuroscience and Center for Molecular Medicine, Karolinska Institutet, Stockholm, Sweden; Department of Medicine, Solna and Center for Molecular Medicine, Karolinska Institutet, Stockholm, Sweden; Department of Clinical Neuroscience and Center for Molecular Medicine, Karolinska Institutet, Stockholm, Sweden; Department of Medicine, Solna and Center for Molecular Medicine, Karolinska Institutet, Stockholm, Sweden/Respiratory Medicine Division, Department of Medicine and Theme Inflammation and Infection, Karolinska University Hospital, Stockholm, Sweden; Department of Medicine, Solna and Center for Molecular Medicine, Karolinska Institutet, Stockholm, Sweden; Department of Medicine, Solna and Center for Molecular Medicine, Karolinska Institutet, Stockholm, Sweden; Institute of Environmental Medicine, Karolinska Institutet, Stockholm, Sweden; Department of Clinical Neuroscience and Center for Molecular Medicine, Karolinska Institutet, Stockholm, Sweden; Department of Clinical Neuroscience and Center for Molecular Medicine, Karolinska Institutet, Stockholm, Sweden; Department of Medicine, Solna and Center for Molecular Medicine, Karolinska Institutet, Stockholm, Sweden; Department of Clinical Neuroscience and Center for Molecular Medicine, Karolinska Institutet, Stockholm, Sweden

**Keywords:** DNA methylation, bronchoalveolar cells, smoking, multiple sclerosis, epigenetics, immunopathogenesis

## Abstract

**Background:**

Despite compelling evidence that cigarette smoking impacts the risk of developing multiple sclerosis (MS), little is known about smoking-associated changes in the primary exposed lung cells of patients.

**Objectives:**

We aimed to examine molecular changes occurring in bronchoalveolar lavage (BAL) cells from MS patients in relation to smoking and in comparison to healthy controls (HCs).

**Methods:**

We profiled DNA methylation in BAL cells from female MS (*n* = 17) and HC (*n* = 22) individuals, using Illumina Infinium EPIC and performed RNA-sequencing in non-smokers.

**Results:**

The most prominent changes were found in relation to smoking, with 1376 CpG sites (adjusted *P* < 0.05) differing between MS smokers and non-smokers. Approximately 30% of the affected genes overlapped with smoking-associated changes in HC, leading to a strong common smoking signature in both MS and HC after gene ontology analysis. Smoking in MS patients resulted in additional discrete changes related to neuronal processes. Methylome and transcriptome analyses in non-smokers suggest that BAL cells from MS patients display very subtle (not reaching adjusted *P* < 0.05) but concordant changes in genes connected to reduced transcriptional/translational processes and enhanced cellular motility.

**Conclusions:**

Our study provides insights into the impact of smoking on lung inflammation and immunopathogenesis of MS.

## Introduction

Multiple sclerosis (MS) is a chronic inflammatory disease of the central nervous system (CNS) characterized by autoimmune damage of myelin sheaths and subsequent neuronal loss. The etiology of MS is complex and largely unknown and influenced by genetic and environmental factors.^1,2^ One of the major environmental risk factors is exposure to tobacco smoke, where daily smokers have an estimated odds ratio (OR) of 1.5 for the risk of developing MS compared to non-smokers.^3^ This risk is being further increased (OR ~ 14) in carriers of the major MS risk variants (HLA-DRB1*15:01+/HLA-A*02−), that is, three times more than risk of haplotype-matched non-smokers.^4,5^ Notably, exposure to cigarette smoke has also been associated with MS disease progression and severity.^1^

Even though the mechanisms underpinning the impact of smoking in MS remain elusive, lung irritation and inflammation induced by exposure to smoke have been proposed to contribute to the immunopathogenesis of MS.^1^ This is supported by a growing body of evidence established in the animal model of MS, experimental autoimmune encephalomyelitis (EAE), presenting the lungs as a site of immune cell priming, prior to CNS infiltration and induction of disease.^6–9^ Accordingly, alveolar macrophages, the first phagocytic cell type exposed to smoke irritants in the pulmonary milieu, have been shown to contribute to induction of adaptive immunity,^10,11^ and EAE pathogenesis.^7,8^ In line with this, smokers exhibit an increased number of alveolar macrophages.^12^

Epigenetic mechanisms, such as DNA methylation, have been proposed to mediate impact of genetic and environmental risk factors in MS. We have previously shown that DNA methylation may mediate genetic risk of HLA-DRB1*15:01 variant in MS through changes in *HLA*-*DRB1* gene expression.^13^ In addition, we have previously found that cigarette smoking not only alters blood DNA methylation in MS patients but also interacts with the disease in a smoking load-dependent manner.^14^ However, so far, little is known about the smoking-associated changes locally, in the primary exposed lung tissue of MS patients.

In this study, we aimed to examine the molecular changes occurring in pulmonary immune cells from MS patients in relation to smoking and in comparison to healthy individuals.

## Material and methods

Full details of experimental procedures are provided in Supplementary Methods.

### Subjects and bronchoscopy

Description of the cohorts used in this study is summarized in Table 1 and details (including smoking, cell proportion, and clinical information) are presented in Supplementary Table 1. Briefly, all participants included in this study were females, 17 MS patients (nine smokers and eight non-smokers), and 22 healthy controls (HCs) (10 smokers and 12 non-smokers), who underwent bronchoscopy with bronchoalveolar lavage (BAL), as previously described.^15^ Healthy volunteers represent a subset of a previously described cohort,^16^ including only women. The study was approved by the Regional Ethical Review Board in Stockholm (Reg. Nos. 2012/1782-31/1 and 2012/1161-31/1) and methods were performed in accordance with institutional guidelines on human subject experiments. All subjects gave their written informed consent.

**Table 1.** Characterization of healthy individuals and multiple sclerosis patients included in our study.

	Multiple sclerosis (MS)		Healthy controls (HCs)	
	Non-smokers	Smokers	Non-smokers	Smokers
Subject	8	9	12	10
Age (years)	43.0 [34.8–49.3]^a^	38.0 [27.0–41.0]	23.5 [22.0–28.5]	28.0 [25.0–37.5]
Cigarettes (day)	N/A	10.0 [6.0–15.0]	N/A	11.3 [10.0–14.4]
Pack (years)	N/A	16.0 [8.0–23.0]^b^	N/A	6.3 [5.5–9.5]
FEV1 (% predicted)	101.5 [95.0–119.3]	106.0 [97.0–112.0]	104.0 [98.0–108.5]	102.5 [90.3–109.3]
FVC (% predicted)	108.0 [90.3–125.0]	114.0 [107.0–126.0]	112.0 [101.8–117.8]	108.0 [101.5–111.0]
FEV/FVC (%)	83.5 [80.0–84.0]	82.0 [71.0–84.0]	82.5 [80.8–84.3]	80.5 [76.5–84.5]
BALF cell concentration (×10^6^/L)	121.6 [96.3–147.1]^a^	371.1 [206.8–476.5]^c^	75.0 [64.1–97.3]	325.8 [226.3–371.3]^d^
BAL recovery (%)	59.0 [57.5–72.0]^a^	54.0 [54.0–66.0]	77.5 [73.3–80.0]	63.5 [58.0–67.8]^d^
BAL macrophages (%)	93.9 [87.9–95.3]	95.6 [94.4–96.6]	90.0 [85.0–93.9]	95.8 [93.7–97.1]^d^
BAL lymphocytes (%)	4.8 [3.8–9.8]	3.0 [2.4–4.4]	8.4 [5.3–11.7]	2.7 [1.9–5.3]^d^
BAL neutrophils (%)	0.9 [0.8–1.3]	0.6 [0.2–0.8]	0.9 [0.4–2.2]	0.8 [0.5–1.2]
BAL eosinophils (%)	0 [0–0]	0.2 [0–0.4]^c^	0 [0–0.3]	0 [0–0.4]
BAL basophils (%)	0 [0–0.05]	0 [0–0]	0 [0–0]	0 [0–0]
BAL mast cells/10 fields of vision	0 [0–1.8]	0 [0–4.0]	0.5 [0–4.5]	2.0 [1.0–3.5]
BAL CD4/CD8 ratio	4.4 [1.2–4.9]	1.4 [0.8–1.9]	1.8 [1.4–2.7]	1.3 [1.0–2.6]

MS: multiple sclerosis; HCs: healthy controls; N/A: not applicable; FEV1: forced expiratory volume in 1 s; FVC: forced vital capacity; BALF: bronchoalveolar lavage fluid; BAL: bronchoalveolar lavage.

Characteristics of female individuals included in our cohort. Data represent *n* or median (25th–75th percentile). Pack (years): (cigarettes smoked per day/20) × years of smoking. Mast cells were counted in 16× magnification.

Statistics calculated using Mann–Whitney U test.

a *P* < 0.05 MS-NS versus HC-NS.

b *P* < 0.05 MS-S versus HC-S.

c *P* < 0.05 MS-S versus MS-NS.

d *P* < 0.05 HC-S versus HC-NS (NS and S indicating non-smoker and smoker, respectively).

### DNA and RNA extraction

Total RNA and genomic DNA were extracted from BAL cells using Allprep DNA/RNA/miRNA universal kit (Qiagen) and quantified by Qubit 3 fluorometer. RNA integrity number (RIN) values were obtained using the RNA 6000 nano chip and the Agilent Bioanalyzer (Agilent Technologies). Samples from MS and controls were processed simultaneously and randomized in downstream analyses.

### DNA methylation analysis

Genomic DNA from BAL cells was processed as previously described,^16^ using the Infinium HumanMethylationEPIC BeadChip Kit (Illumina). MS and HC samples were randomized according to smoking status, age, gender, and cell proportion and processed together with technical replicates in one run. We assessed DNA methylation (5mC), DNA hydroxymethylation (5hmC), and bulk DNA modifications (total 5mC + 5hmC) by performing in parallel bisulfite (BS) and oxidative BS treatments of genomic DNA, respectively. Briefly, BS *β*-values represent total 5mC + 5hmC, while oxBS *β*-values reflect 5mC. Hydroxymethylation values were generated by subtracting normalized oxBS from BS *β*-values, as previously described.^16^ BS and oxBS samples from each individual were processed together and run on the same array. Raw IDAT files were processed as previously described (Supplementary Figure 1).^16^ Only probes (734,078) and samples (*n* = 39) shared between BS and oxBS datasets were included in subsequent analyses. We used limma to fit a linear model to each methylation *M*-value (transformed *β*-value) and empirical Bayes to calculate test statistics for group comparisons, adjusting for covariates (age and macrophage fraction). Multiple testing correction was performed by adjusting the false discovery rate (FDR) and was based on all included probes (*n* = 734,078) for each group comparison. Probes were considered significantly differentially methylated between groups when the Benjamini–Hochberg-adjusted (BH-*P*_adj_) < 0.05. Statistical analysis of enriched and depleted differential methylation was performed using Pearson’s Chi-square test on contingency tables of count data, and *P* values were adjusted for multiple testing (*n* = 8) using Bonferroni.

### Gene expression analysis

cDNA libraries were prepared by poly-A capture of 150 ng purified total RNA from each individual, using a modified version of SMART-seq2 to adjust for bulk input,^17^ and subsequently sequenced at 125 bp paired-end on an Illumina HiSeq 2500. RNA-sequencing reads were subjected to quality filtering, adapter trimming using in Trim Galore with default parameter settings, and aligned to the transcriptome using the pseudoalignment-based Kallisto algorithm. For downstream analysis, only females and groups including a minimum of seven samples with an RIN value above seven were included. Genes with >10 normalized read counts were kept. A total of 15 samples from non-smoker individuals passed these criteria, that is, seven MS and eight HC, which were used for differential expression analysis using DESeq2 package in R. We adjusted for the covariate age and considered transcripts with unadjusted *P* value < 0.05 suitable for gene ontology (GO) analysis.

### GO analyses

GO analyses were performed using ingenuity pathway analysis (IPA; Qiagen) of the annotated differentially methylated and expressed genes, using unbiased parameters for all criteria and right-tailed Fisher’s exact test *P* values < 0.05 were considered statistically significant. We confirmed IPA results using over-representation analysis (ORA).^18^ Visualization of genes and GO terms was performed using STRING database version 10.5 and REVIGO tool.^19^

### Data availability

DNA methylation data from this study is available in Gene Expression Omnibus (GEO) database (under accession number GSE151017) and the RNA-sequencing data are available upon request.

## Results

### Smoking-associated DNA methylation changes in BAL cells from MS patients

**Figure 1.**
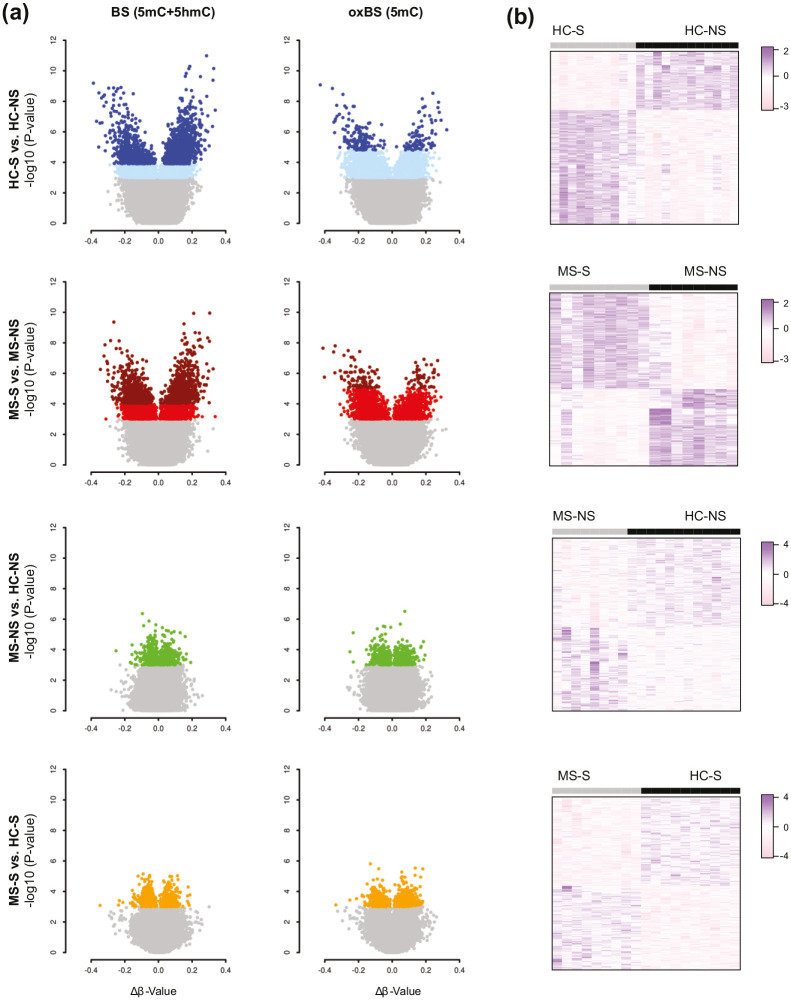
Smoking-associated methylation changes in bronchoalveolar cells from multiple sclerosis (MS) patients and healthy controls (HC). (a) Volcano plots illustrating, from top to bottom, differences in DNA methylation between smokers and non-smokers in HC (HC-S vs. HS-NS, blue) and in MS patients (MS-S vs. MS-NS, red) and between MS patients and HC, distinguishing non-smokers (MS-NS vs. HC-NS, green) and smokers (MS-S vs. HC-S, orange). Hyper- and hypomethylated CpGs with BH-*P*_adj_ < 0.05 are indicated in dark colors, while lighter colors represent CpGs with unadjusted *P* value < 0.001. (b) Heatmaps were generated using the 1000 most significant differentially methylated CpGs between the conditions (the scale represents *z*-score transformed *β*-values). Full data are provided in Supplementary Table 2.

We characterized total BS, 5mC and 5hmC changes in BAL cells of female MS patients (*n* = 17), and HC (*n* = 22) (Table 1, Supplementary Table 1) using Illumina HumanMethylationEPIC arrays. We examined the impact of smoking (S) compared to non-smoking (NS) within each group (MS-S vs. MS-NS and HC-S vs. HC-NS) and compared MS patients to HCs for both smokers and non-smokers, separately (MS-NS vs. HC-NS and HC-S vs. MS-S). Analysis identified 1376 BS, 131 5mC, and 4 5hmC differentially methylated positions (DMPs) associated with smoking in MS patients (MS-S vs. MS-NS) after correction for confounders (Benjamini–Hochberg-adjusted (BH-*P*_adj_) < 0.05) (Figure 1). The 10 most significant DMPs are listed in Table 2 (full data are shown in Supplementary Table 2). DMP analysis in healthy individuals yielded higher number of smoking-associated DMPs, with 1821 BS-DMPs, 234 5mC-DMPs, and one 5hmC-DMP identified between smokers and non-smokers (BH-*P*_adj_ < 0.05). Notably, about one-third of the BS-DMPs (491/1376) and 5mC-DMPs (41/131) found associated to smoking in MS overlapped with DMPs identified in HC, with comparable effect size represented by Δ*β* values (Supplementary Figure 2). Overall, the effect size at overlapping and non-overlapping BS-DMPs displayed strong correlation (*r* = 0.8, *P* < 2.2E–16) between MS and HC, with, as expected, magnitude of change mirroring statistical differences, that is,|Δ*β*_MS_| >|Δ*β*_HC_| at smoking-associated BS-DMPs identified in MS and not in HC and vice versa (Supplementary Figure 2). Overlapping DMPs between MS and HC maps to well-known smoking-associated CpGs, such as *AHRR, RARA*, or *HIVEP3*.^16,20,21^ Around half of the 5mC-DMPs (64/131 in MS and 138/234 in HC) are overlapped with BS-DMPs in each group (Supplementary Figure 2). No significant DMPs were associated with MS compared to controls in the smoker or non-smoker groups after correction for multiple testing (Figure 1).

**Table 2.** Top 10 significant smoking-associated DMPs of BS methyl (5mC + 5hmC), true 5mC (oxBS), and 5hmC in MS patients (MS-S vs. MS-NS).

Probe	*P* value	Adj. *P* value	Chr	Position	Gene	Δ*β*	Island_Rel	Feature	Enhancer
*BS*									
* cg12703333*	1.1E−10	4.3E–05	chr10	76962548		0.30	OpenSea	IGR	
* cg09020840*	1.2E−10	4.3E–05	chr17	73087391	*SLC16A5*	0.21	S_Shelf	5ʹUTR	
* cg22145459*	4.3E−10	1.0E–04	chr6	31693656	*C6orf25*	−0.26	S_Shore	3ʹUTR	
* cg11823253*	5.7E−10	1.0E–04	chr1	213154553	*VASH2*	0.15	OpenSea	Body	
* cg01034754*	2.1E−09	2.6E–04	chr10	30720817		0.22	N_Shore	IGR	
* cg06275684*	2.3E–09	2.6E–04	chr12	65074198	*RASSF3*	0.24	OpenSea	Body	*
* cg24439401*	2.4E–09	2.6E–04	chr3	16282260		0.15	OpenSea	IGR	
* cg26744946*	4.6E–09	3.9E–04	chr8	145065833	*GRINA*	0.16	S_Shore	Body	
* cg11650372*	4.7E–09	3.9E–04	chr21	35171932	*ITSN1*	0.29	OpenSea	Body	
* cg07249224*	6.0E–09	3.9E–04	chr16	30101680	*TBX6*	0.25	N_Shore	Body	
*5mC*									
* cg10655682*	1.6E–08	5.8E–03	chr19	4567177		−0.34	S_Shore	IGR	*
* cg24699021*	2.2E–08	5.8E–03	chr5	172305986	*ERGIC1*	−0.41	OpenSea	Body	
* cg22145459*	3.2E–08	5.8E–03	chr6	31693656	*C6orf25*	−0.26	S_Shore	3ʹUTR	
* cg20472746*	3.7E–08	5.8E–03	chr13	42039334	*C13orf15*	−0.19	OpenSea	Body	
* cg14223856*	4.0E–08	5.8E–03	chr9	139508740		−0.35	OpenSea	IGR	
* cg07457727*	6.6E–08	8.0E–03	chr8	131451983		−0.29	N_Shelf	IGR	
* cg01668352*	8.6E–08	8.0E–03	chr12	64482597	*SRGAP1*	−0.23	OpenSea	Body	
* cg24790419*	8.8E–08	8.0E–03	chr19	18385930	*KIAA1683*	−0.24	OpenSea	TSS1500	
* cg01233673*	1.2E–07	9.5E–03	chr18	33517119		0.19	OpenSea	IGR	
* cg25468274*	1.3E–07	9.5E–03	chr1	31280338		−0.22	Island	IGR	
*5hmC*									
* cg18513023*	6.1E–08	2.9E–02	chr6	44009266		0.13	OpenSea	IGR	*
* cg13297582*	1.0E–07	2.9E–02	chr18	13288627	*LDLRAD4*	0.08	OpenSea	5ʹUTR	
* cg19800026*	1.5E–07	2.9E–02	chr5	14492945	*TRIO*	0.21	OpenSea	Body	
* cg22944934*	1.6E–07	2.9E–02	chr6	31621765	*BAT3*	0.17	S_Shore	TSS1500	
* cg14341968*	9.6E–07	1.4E–01	chr9	136075868		0.04	S_Shore	IGR	
* cg26650480*	1.2E–06	1.4E–01	chr20	4796176	*RASSF2*	0.11	OpenSea	5ʹUTR	
* cg09655482*	1.3E–06	1.4E–01	chr10	27131072	*ABI1*	0.11	OpenSea	Body	
* cg03964696*	1.6E–06	1.5E–01	chr2	74734085	*PCGF1*	0.21	N_Shore	Body	
* cg22502837*	2.2E–06	1.8E–01	chr12	56137111	*GDF11*	0.03	Island	1stExon	
* cg19629631*	4.3E–06	3.1E–01	chr7	2060116	*MAD1* *L1*	0.12	Island	Body	*

DMPs: differentially methylated positions; BS: bisulfate; MS: multiple sclerosis; S: smoker; NS: non-smoker; IGR: intergenic region; Probe: Illumina probe ID; Adj. *P* value: Benjamini–Hochberg corrected *P* value (FDR); Chr: chromosome; Gene: UCSC gene name; Δ*β*: difference in mean *β*-values between smokers and non-smokers; Island_Rel: relation to CpG Island; Feature: gene feature; Enhancer: identified enhancer in FANTOM5 consortium; *annotated as enhancer.

One BS-DMP mapping to the *AHRR/EXOC3* locus (cg25648203, BH-*P*_adj_ < 0.05) overlapped with smoking-associated DMPs previously reported in whole blood from MS patients.^14^ Interestingly, cg25648203 displayed increased effect size in BAL cells compared to blood (Δ*β*_BAL_ = −0.251 vs. Δ*β*_blood_ = −0.052; Supplementary Table 3). Comprehensive comparison of the impact of smoking in blood and BAL revealed that overall BAL cells displayed larger magnitude of changes compared to blood (Supplementary Figure 3). Increased effect size in BAL cells compared to blood was visible at all probes shared by BAL and blood (*n* = 382,277) (median BAL vs. blood: 0.12 vs. 0.03 and −0.13 vs. −0.03, for hypermethylated and hypomethylated sites, respectively) and was particularly apparent at hypomethylated BS-DMPs in blood (*n* = 41, FDR < 0.05) (median BAL vs. blood: −0.45 vs. −0.28). Expectedly, significant positive correlation of effect size was observed between significant blood DMPs (*n* = 50, FDR < 0.05) and the corresponding CpGs in BAL. On the contrary, no significant correlation was observed between BS-DMPs in BAL (BH-P_adj_ < 0.05) and the corresponding CpGs in blood (*n* = 507), suggesting that the primarily exposed BAL macrophages display a unique signature that might be diluted in blood tissue. Unsurprisingly, these BAL BS-DMPs displayed larger effect size in BAL compared to blood (*P* < 2.2E-16) at both hypomethylated (median BAL vs. blood: −0.99 vs. −0.03,) and hypermethylated (median BAL vs. blood: 0.85 vs. 0.03) sites (Supplementary Figure 3).

Altogether, these findings indicate that overall BAL cells from MS patients and HC individuals display highly similar methylome. Smoking exerts a prominent impact on the methylome of BAL cells in both MS patients and HCs.

### Gene-related smoking-associated differences between MS patients and healthy individuals

**Figure 2.**
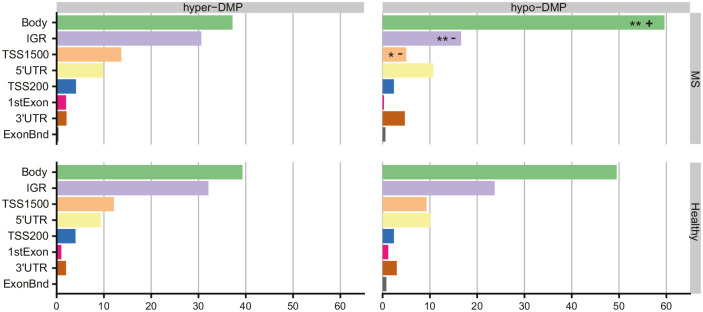
Distribution of smoking-associated hyper- and hypomethylated CpGs in multiple sclerosis (MS) patients and healthy controls (HCs) across gene features. Horizontal bar plots representing relative frequencies of hyper- and hypomethylated BS-DMPs associated with smoking in MS and HC, shown as percentage (%) across gene features (TSS1500, TSS200, 1stExon, 5ʹUTR, Body, 3ʹUTR, ExonBnd, and IGR). Enrichment/depletion analysis was performed using chi-square test on frequencies, with *P* values adjusted for multiple testing (Bonferroni). *Bonferroni-*P*_adj_ < 0.05 compared to smoking-associated BS-DMP profile in HC **Bonferroni-*P*_adj_ compared to smoking-associated BS-DMP profile in HC. Enriched features are represented by a positive symbol (+) and depleted features are represented by a negative symbol (−).

To determine genome-wide differences in DNA methylation smoking profiles between MS and HC in BAL cells, we stratified the significant BS-DMPs according to gene-related features and performed enrichment/depletion analysis. In both MS and HC groups, smoking-associated BS-DMPs were significantly enriched in gene bodies (Bonferroni-adjusted chi-square *P*_adj_ = 7.5 × 10^−18^ and *P*_adj_ = 1.1 × 10^−8^, respectively) while depleted at TSS200 (200 bp-segment upstream the transcription starting site; *P*_adj_ = 1.0 × 10^−10^ and *P*_adj_ = 1.3 × 10^−13^, respectively) and first Exon (*P*_adj_ = 6.0 × 10^−05^ and *P*_adj_ = 8.8 × 10^−7^, respectively), compared to the probes distribution of the EPIC array (734,078) (Supplementary Figure 4). However, significant depletion of BS-DMPs in TSS1500 was observed in MS patients (*P*_adj_ = 0.0037 and *P*_adj_ = 0.25 for MS and HC, respectively). Differences between MS and HC were minor, that is, showing fewer DMPs in intergenic regions (IGRs) in MS compared to controls (*P*_adj_ = 0.017, Supplementary Figure 4). To address whether this finding was due to varying hyper- or hypo-methylation changes across features, we further stratified the BS-DMPs into hyper- and hypomethylated sites (Figure 2). Analysis of smoking-associated hypermethylated BS-DMPs showed no significant difference between the smoking-associated signatures of MS patients and HC. In contrast, hypomethylated BS-DMPs were found enriched in gene bodies (*P*_adj_ = 0.0013) and depleted in IGR and TSS1500 (*P*_adj_ = 0.0096 and *P*_adj_ = 0.017, respectively) in MS patients compared to HC.

### Common and distinct smoking signatures in MS patients and healthy individuals

**Figure 3.**
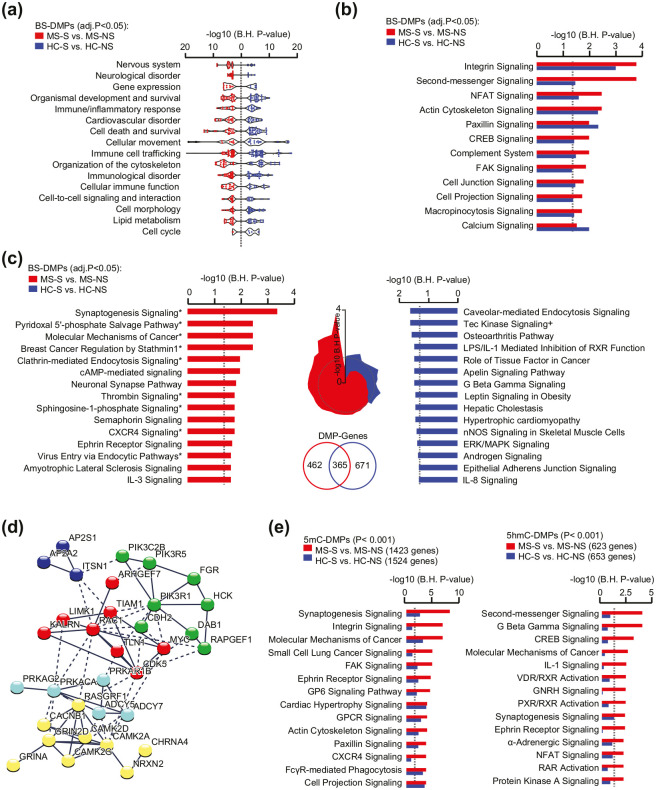
Functional annotations of genes comprising smoking-associated CpGs in multiple sclerosis (MS) patients and healthy controls (HCs). (a) Enriched categories of *Diseases and Biological Functions* related to BS-DMP genes (BH-*P*_adj_ < 0.05) associated with smoking in MS and HC, depicted in red and blue colors, respectively. Of note, terms linked to *Cancer* were excluded from the visualization. (b) Shared canonical pathways related to BS-DMP (BH-*P*_adj_ < 0.05) associated with smoking in MS patients (red) and HC individuals (blue). (c) Top canonical pathways related to BS-DMP (BH-*P*_adj_ < 0.05) genes associated with smoking in MS patient (left panel) or HC (right panel), specifically. Radar chart (middle) indicates the overlap between all specific terms and the dotted line representing the significance threshold. Significant enrichment for BS-DMP genes (BH-*P*_adj_ < 0.05) identified in MS patients or in HC individuals and not overlapping between the groups are indicated with * or +, respectively, with a Venn diagram numbering the genes. (d) Representation of genes from the *Synaptogenesis signaling* pathway network using STRING analysis. Gray gradient indicates the strength of data support (darker gray representing stronger evidence) and colors reflect different cluster (kmeans clustering set at 5 clusters). (e) Canonical pathways related to candidate 5mC-DMPs (left panel) and 5hmC-DMPs (right panel) (unadjusted *P* < 0.001) associated with smoking in MS patients (red) and HC individuals (blue). For all enrichment analyses, significance is represented as −log10 *P* value after adjustment using Benjamini–Hochberg (BH) correction obtained with ingenuity pathway analysis. Full data are presented in Supplementary Table 4.

To gain insight into the functional relevance of changes associated with smoking in MS patients, we performed GO analyses of the 827 annotated genes associated with smoking (1376 BS-DMPs, BH-*P*_adj_ < 0.05) in MS patients (MS-S vs. MS-NS) as well as of the 1036 genes (1821 BS-DMPs, BH-*P*_adj_ < 0.05) altered after smoking in healthy individuals (HC-S vs. HC-NS). Findings from IPA uncovered a strong smoking-associated signature shared by smokers in both groups compared to respective non-smokers (Supplementary Table 4). Enrichment analysis for *Biological Functions and Diseases* showed that, despite limited overlap on the DMP level, most categories are represented in both MS and HC with the top significant terms (outside cancer-related terms) connected to cellular movement, organization of the cytoskeleton, and immune trafficking (Figure 3(a)). Subtle smoking-related differences between MS and HC groups include more pronounced enrichment for terms related to the nervous system/neurological diseases, cell death/survival, and gene expression in MS patients and lipid metabolism and cell cycle in HC (Supplementary Table 4). Both groups also shared smoking-associated gene enrichment of *Canonical Pathways* involved in cellular adhesion and migration such as integrin, actin cytoskeleton, and paxillin signaling (Figure 3(b)). Examination of pathways that are specifically enriched in either MS or HC group after smoking revealed a striking “neuronal” signature in MS patients, with synaptogenesis signaling pathway as the most enriched process, contrasting with the top canonical immune-related pathways altered by smoking in HC (Figure 3(c)). This finding was further supported by IPA of non-overlapping DMP genes that were found altered by smoking in MS patients but not in HC (*n* = 462 genes, BH-*P*_adj_ < 0.05, Figure 3(c)). “Synaptic” process implicated a network of functionally interconnected genes encoding leukocyte adhesion molecules (e.g. *NRXN2, CDH2*, and *TLN1*), clathrin-associated molecules (*AP2S1, AP2A1*, and *ITSN1*), glutamate and nicotinic cholinergic receptor subunits (i.e. *GRINA, GRIN2D*, and *CHRNA4*), as well as downstream signaling molecules such as protein kinases A, RhoGTases (e.g. *RAC1* and *TIAM1*), among others (Figure 3(d)). GO analysis of 5mC or 5hmC candidate DMPs (unadjusted *P* < 0.001) confirmed enrichment of *canonical pathways* related to cellular motility and immune processes in relation to smoking in both groups, while stronger enrichment of nervous-related terms could be observed in MS-S vs. MS-NS (Figure 3(e), Supplementary Table 4). These alterations are shared by overlapping and non-overlapping BS, 5mC, or 5hmC DMPs (Supplementary Figure 5).

Altogether, these data suggest that in addition to altering biological processes that are common to healthy individuals, for example, cytoskeleton rearrangement and cellular movement, smoking induces minor distinct changes in MS patients.

### Subtle changes between MS patients and healthy individuals implicate altered transcriptional machinery and enhanced cellular motility

**Figure 4.**
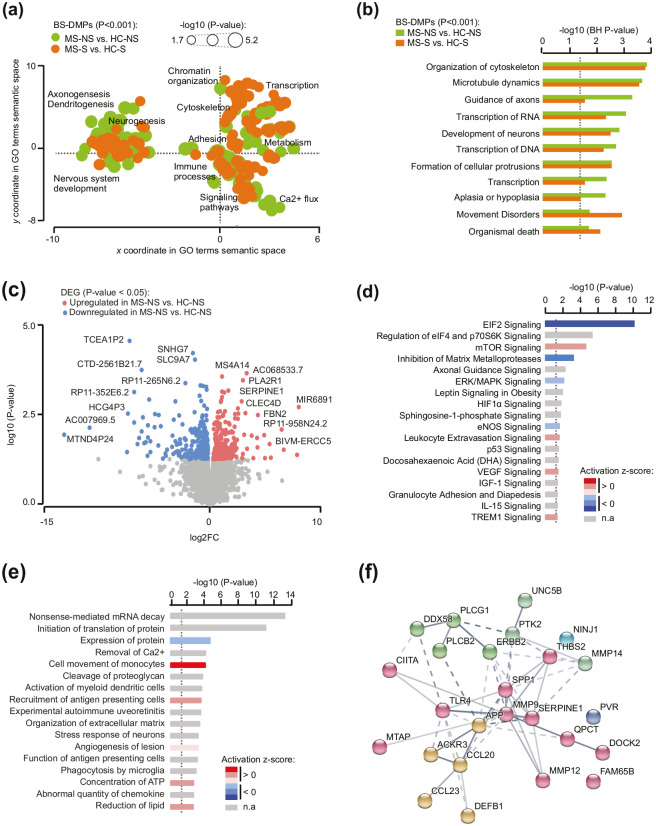
Functional annotation of methylation and transcriptional changes in multiple sclerosis (MS) patients compared to healthy controls (HCs). (a) Scattered plot showing clusters of gene ontology (GO) terms associated with differentially methylated CpGs (unadjusted *P* < 0.001) in MS patients compared to HC, in both non-smokers (green) and smokers (orange). GO terms were obtained using over-representation analysis and clusters were visualized in two-dimensional space (assigning *x* and *y* coordinates to each term) by applying multidimensional scaling of the matrix of GO terms according to semantic similarity, using REVIGO. The circle size represents −log10 (*P* value), with small to big diameters ranging from 1.76 (*P* = 0.01) to 5.17 (*P* = 6.8 × 10^−06^). (b) Top shared *Biological processes and Disease* for differentially methylated CpGs in MS patients compared to HC, in both non-smokers (green) and smokers (orange), obtained using ingenuity pathway analysis (IPA). (c) Volcano plot illustrating the differentially expressed genes between MS and HC non-smokers. Red and blue indicate upregulated and downregulated, respectively, genes in MS compared to HC at unadjusted *P* < 0.05 (for full data, see Supplementary Table 5). Top canonical pathways (d) and *Biological functions and Diseases* (e) associated with differentially expressed genes, obtained using IPA. Significance is represented as −log10 (*P* value) and colors indicate predicted activation z-score, with decreased and increased activity in blue and red, respectively. n.a. denotes prediction not available. (f) Representation of genes network from the term Cell movement of mononuclear leukocytes using STRING analysis. Gray gradient indicates the strength of data support (darker gray representing stronger evidence) and colors reflect different clusters (kmeans clustering set at 5). Full data from IPA are provided in Supplementary Table 4.

In order to elucidate MS-specific changes, we focused on the functional relevance of DNA methylation changes in MS patients in comparison to healthy individuals (MS-NS vs. HC-NS and MS-S vs. HC-S). This approach is undeniably exploratory as no significant changes could be detected between MS patients and HC individuals, disregarding the smoking status, after correction for multiple testing (Figure 1). Nevertheless, GO analyses conducted on candidate BS-DMPs (unadjusted *P* < 0.001) uncovered common MS-associated alterations in both smokers and non-smokers, which converged to three major processes: cytoskeleton dynamics and cellular mobility, RNA expression, and neuronal processes (Figure 4(a) and (b)). These functions were also significantly enriched in genes harboring 5mC and 5hmC changes (unadjusted *P* < 0.001) (Supplementary Table 4). To further decipher these findings, we conducted transcriptome analysis (RNA-sequencing) in BAL cell samples. Sufficient amount and quality of RNA was found in non-smoker MS (*n* = 7) and HC (*n* = 8). Analysis identified that a total of 487 transcripts were differentially expressed in MS patients compared to HC at a unadjusted *P* value < 0.05 (Figure 4(c), Supplementary Table 5). None of these genes remained significantly associated after adjustment for multiple testing (BH-*p*_adj_ < 0.05), confirming that BAL cells from MS patients display only tenuous differences compared to healthy individuals in our cohort. Nevertheless, GO analyses on putative candidate genes (unadjusted *P* < 0.05) revealed prominent changes of the transcriptional and translational machinery with reduced activity of eukaryotic translation initiation factor EIF2 signaling as the top *Canonical Pathways* altered in MS patients compared to controls (Figure 4(d)). Analysis of *Biological Functions and Diseases* further corroborated this finding with enrichment of mRNA decay and reduced protein expression (Figure 4(e)). Alteration of global transcription was driven by slight downregulation (logFC > −1) of a plethora of ribosomal subunits along with EIF2/F4 and Argonaute 1 (*AGO1*) genes (Supplementary Table 4). In addition, GO findings implicated an increased activity of cellular processes related to cell movement of monocytes, leukocyte extravasion, recruitment of antigen presenting cells, and disinhibition of matrix metalloproteases (MMPs) (Figure 4(e)). Gene network associated to these processes involved potent dysregulation (logFC > 1) of immune mediators such as chemokines (*CCL23, CCL20*, and *ACKR3*), osteopontin (*SPP1*), toll-like receptor 4 (*TLR4*), HLA class II transactivator (*CIITA*), defensin (*DEFB1*), amyloid *β* precursor (*APP*), as well as cell adhesion and migration molecules (*NINJ1, SERPINE1, UNC5B, THBS2*, and several *MMP* genes) (Figure 4(f)).

Overall, methylome and transcriptome analyses suggest that BAL cells from MS patients display very subtle (not reaching the significance threshold after statistical adjustment) but concordant changes connected to reduced transcriptional/translational machinery and enhanced cellular motility.

## Discussion

We examined the molecular changes occurring in BAL cells from MS patients, distinguishing smokers from non-smokers and in comparison with healthy individuals. Overall, BAL cells from MS patients exhibited very similar changes compared to HC individuals. Genome-wide methylome analysis revealed a potent impact of cigarette smoking on BAL cells from MS patients, with a signature that strongly resembles the one found in healthy individuals. In addition to these shared changes, smoking in MS patients is associated with modest epigenetic changes affecting genes that are seemingly related to nervous processes. Findings from combined methylome and transcriptome data suggest that BAL cells from MS patients exhibit tenuous alterations converging to reduced transcriptional/translational machinery and increased cellular motility.

The most prominent changes were detected in relation to smoking. Notably, BS-DMPs are also known smoking-associated DMPs in healthy individuals,^21^ for example, several sites annotated to the aryl hydrocarbon receptor repressor (*AHRR*) gene. *AHRR* expression induced by carcinogenic polycyclic aromatic hydrocarbons, which are increased in the lung tissue of smokers,^22^ associates with *AHRR* DNA methylation in blood,^14,23^ lung tissue,^24^ and BAL cells.^16^ Some of the identified BS-DMPs in MS BAL cells were also found significantly associated with smoking in blood from MS patients.^14^ Interestingly, smoking exerted a stronger impact in BAL cells compared to blood at most overlapping DMPs, for example, at *AHRR/EXOC3* locus. This is consistent with a study examining *AHRR* locus in BAL macrophage and peripheral leukocytes from healthy individuals^23^ and suggests that the greater amplitude of changes observed in the primarily affected lung tissue reflects its proximity to smoke exposure. Smoking-associated effects on DNA methylation at *AHRR* in MS patients could be of high relevance, since the presence of MS disease can modify the smoking-associated effects on DNA methylation at this gene.^14^

Interestingly, functional annotation of the smoking-associated DMPs found in MS revealed a significant enrichment of pathways connected to neuronal processes such as axonal guidance and synaptic plasticity, a seemingly peculiar pattern in BAL immune cells. These pathways indeed overlap with the molecular alterations identified in neurons from MS patients, *post*-*mortem.*^25^ Of note, most of them have been shown to participate in immune processes outside of the nervous system. For instance, glutamate N-methyl-D-aspartate (NMDA) receptor (*GRINA, GRIN2D*), which are constitutively expressed by immune cells, can mediate lung injury caused by oxidative stress,^26,27^ and are required for myeloid cells to shape T cell response in the context of MS-like disease EAE.^28^ Nicotinic AChR subunit encoded by *CHRNA4* exhibits a strong non-neuronal expression pattern correlating with genes involved in the nicotine metabolism,^29^ and genetic variation in *CHRNA4* has been associated with smoking-related diseases.^30,31^ Consistent with this, GO analysis of 5mC or 5hmC changes replicated findings from BS-DMPs, with enrichment of functions pertaining to cellular movement, intracellular trafficking and immune processes as well as neuronal processes. This can be exemplified with 5hmC-DMPs (*P_adj_* < 0.05) mapping to BAG6 (*BAT3*) and *LDLRAD4* promoters. *BAG6* encodes a pro-apoptotic gene and *LDLRAD4* encodes a transforming growth factor (TGF)-*β* signaling inhibitor dysregulated upon exposure to a constituent of cigarette smoke.^32^ Interestingly, the positive correlation between BS-Δ*β* and 5mC-Δ*β* or 5hmC-Δ*β* (Supplementary Figure 5) suggests that BS-based differences primarily reflect strong changes in either 5mC or 5hmC or small differences in both modifications. On the contrary, the anti-correlated 5mC and 5hmC changes at CpGs (*P* < 0.001) that were not identified as BS-DMPs implies that a minor fraction of smoking-associated changes, likely arising from inflammation-induced oxidation, might escape detection using BS-based methodology, as previously reported.^16^ While the mechanisms underlying this incongruous pattern in BAL cells and its functional consequences remain to be explored, it is noteworthy that a similar signature was found in blood immune cells from progressive MS patients and not in patients in the early relapsing-remitting phase of disease.^33^ Knowing that smoking not only increases the risk to develop MS but also affects its progression and severity, one can speculate that the differences observed in BAL cells from MS smokers compared to non-smokers might contribute to unfavorable evolution of disease. In line with this hypothesis, smoking-associated DNA methylation changes hold a strong predictive value for poorer cognitive performance, brain structural integrity and psychophysical health.^34^

We further sought to characterize the intrinsic profile of BAL cells from MS patients compared to HCs. Both methylome and transcriptome profiling in non-smokers resulted in limited changes between MS and HC individuals, insofar as no DMPs or transcripts passed significance after adjustment for multiple testing. This could be driven by minor biological differences between MS and HC non-smokers or due to the lack of power to detect such changes in a relatively small and heterogeneous cohort. Nevertheless, GO analyses of the affected genes at a nominal significance threshold unraveled coherent changes reflecting hampered transcriptional and translational processes and enhanced migratory ability in BAL cells of MS patients, the latter being driven by upregulation of several pro-inflammatory genes, MS-associated genes (e.g. osteopontin), and adhesion/migration molecules such as MMPs and Ninjurin 1. Despite the undeniable lack of power in our study and the necessary caution in drawing conclusions from alterations observed primarily at the GO level, the results obtained in human BAL cells in the context of MS corroborate the molecular signature found in animal studies of EAE.^6^ In EAE animals, lung-mobilized immune cells attain enhanced mobility through intense gene reprogramming in the lungs, marked by downregulation of their activation program and upregulation of cellular locomotion transcripts, with a pivotal role of Ninjurin 1. By shedding light on potentially similar mechanisms in lung immune cells from MS patients, our study provides translational insight into putative pulmonary mechanisms favoring immune cell encephalitogenicity in MS.

Thus, our findings indicate that while BAL cells from MS patients display comparable molecular changes compared to healthy individuals, slight changes could be detected between the groups in the absence or presence of smoke exposure. The findings support the hypothesis of a relationship between the lungs and the CNS in the context of autoimmunity and might contribute to a better understanding of MS pathology. This is particularly important in light of the potential of lifestyle interventions in the prevention and mitigation of smoking in general and in MS more specifically.

## Supplemental Material

MSJ943768_supplemental_figures – Supplemental material for Methylome and transcriptome signature of bronchoalveolar cells from multiple sclerosis patients in relation to smoking Click here for additional data file. Supplemental material, MSJ943768_supplemental_figures for Methylome and transcriptome signature of bronchoalveolar cells from multiple sclerosis patients in relation to smoking by Mikael V Ringh, Michael Hagemann-Jensen, Maria Needhamsen, Susanna Kullberg, Jan Wahlström, Johan Grunewald, Boel Brynedal, Maja Jagodic, Tomas J Ekström, Johan Öckinger and Lara Kular in Multiple Sclerosis Journal

MSJ943768_supplemental_material – Supplemental material for Methylome and transcriptome signature of bronchoalveolar cells from multiple sclerosis patients in relation to smoking Click here for additional data file. Supplemental material, MSJ943768_supplemental_material for Methylome and transcriptome signature of bronchoalveolar cells from multiple sclerosis patients in relation to smoking by Mikael V Ringh, Michael Hagemann-Jensen, Maria Needhamsen, Susanna Kullberg, Jan Wahlström, Johan Grunewald, Boel Brynedal, Maja Jagodic, Tomas J Ekström, Johan Öckinger and Lara Kular in Multiple Sclerosis Journal

MSJ943768_supplemental_methods – Supplemental material for Methylome and transcriptome signature of bronchoalveolar cells from multiple sclerosis patients in relation to smoking Click here for additional data file. Supplemental material, MSJ943768_supplemental_methods for Methylome and transcriptome signature of bronchoalveolar cells from multiple sclerosis patients in relation to smoking by Mikael V Ringh, Michael Hagemann-Jensen, Maria Needhamsen, Susanna Kullberg, Jan Wahlström, Johan Grunewald, Boel Brynedal, Maja Jagodic, Tomas J Ekström, Johan Öckinger and Lara Kular in Multiple Sclerosis Journal

REVISED_Supplementary_Table_2_light – Supplemental material for Methylome and transcriptome signature of bronchoalveolar cells from multiple sclerosis patients in relation to smoking Click here for additional data file. Supplemental material, REVISED_Supplementary_Table_2_light for Methylome and transcriptome signature of bronchoalveolar cells from multiple sclerosis patients in relation to smoking by Mikael V Ringh, Michael Hagemann-Jensen, Maria Needhamsen, Susanna Kullberg, Jan Wahlström, Johan Grunewald, Boel Brynedal, Maja Jagodic, Tomas J Ekström, Johan Öckinger and Lara Kular in Multiple Sclerosis Journal

REVISED_Supplementary_Table_4 – Supplemental material for Methylome and transcriptome signature of bronchoalveolar cells from multiple sclerosis patients in relation to smoking Click here for additional data file. Supplemental material, REVISED_Supplementary_Table_4 for Methylome and transcriptome signature of bronchoalveolar cells from multiple sclerosis patients in relation to smoking by Mikael V Ringh, Michael Hagemann-Jensen, Maria Needhamsen, Susanna Kullberg, Jan Wahlström, Johan Grunewald, Boel Brynedal, Maja Jagodic, Tomas J Ekström, Johan Öckinger and Lara Kular in Multiple Sclerosis Journal

Supplementary_Table_1_Ringh_Hagemann-Jensen_et_al – Supplemental material for Methylome and transcriptome signature of bronchoalveolar cells from multiple sclerosis patients in relation to smoking Click here for additional data file. Supplemental material, Supplementary_Table_1_Ringh_Hagemann-Jensen_et_al for Methylome and transcriptome signature of bronchoalveolar cells from multiple sclerosis patients in relation to smoking by Mikael V Ringh, Michael Hagemann-Jensen, Maria Needhamsen, Susanna Kullberg, Jan Wahlström, Johan Grunewald, Boel Brynedal, Maja Jagodic, Tomas J Ekström, Johan Öckinger and Lara Kular in Multiple Sclerosis Journal

Supplementary_Table_3_Ringh_Hagemann-Jensen_et_al – Supplemental material for Methylome and transcriptome signature of bronchoalveolar cells from multiple sclerosis patients in relation to smoking Click here for additional data file. Supplemental material, Supplementary_Table_3_Ringh_Hagemann-Jensen_et_al for Methylome and transcriptome signature of bronchoalveolar cells from multiple sclerosis patients in relation to smoking by Mikael V Ringh, Michael Hagemann-Jensen, Maria Needhamsen, Susanna Kullberg, Jan Wahlström, Johan Grunewald, Boel Brynedal, Maja Jagodic, Tomas J Ekström, Johan Öckinger and Lara Kular in Multiple Sclerosis Journal

Supplementary_Table_5_Ringh_Hagemann-Jensen_et_al – Supplemental material for Methylome and transcriptome signature of bronchoalveolar cells from multiple sclerosis patients in relation to smoking Click here for additional data file. Supplemental material, Supplementary_Table_5_Ringh_Hagemann-Jensen_et_al for Methylome and transcriptome signature of bronchoalveolar cells from multiple sclerosis patients in relation to smoking by Mikael V Ringh, Michael Hagemann-Jensen, Maria Needhamsen, Susanna Kullberg, Jan Wahlström, Johan Grunewald, Boel Brynedal, Maja Jagodic, Tomas J Ekström, Johan Öckinger and Lara Kular in Multiple Sclerosis Journal
