# Supplementary material for: Methylome and transcriptome signature of bronchoalveolar cells from multiple sclerosis patients in relation to smoking
Source: Mult Scler. 2020 Jul 30;27(7):1014–26. doi: 10.1177/1352458520943768 (PMC8145441; doi:10.1177/1352458520943768)
Supplement: MSJ943768_supplemental_figures – Supplemental material for Methylome and transcriptome signature of bronchoalveolar cells from multiple sclerosis patients in relation to smoking [file MSJ943768_supplemental_figures.pdf]

Supplementary Figure 1

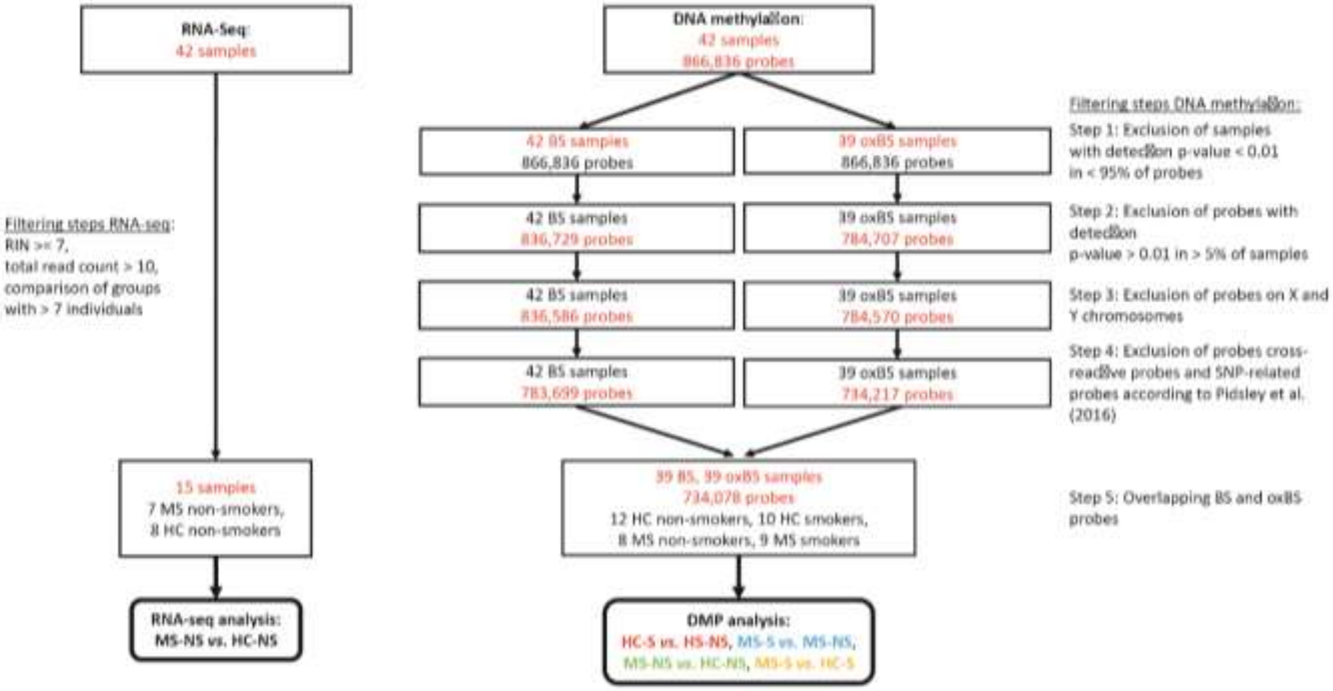

Supplementary Figure 1. Analytical workflow

The schematic is representing the filtering steps performed before statistical analyses of RNA-seq and DNA methylation/hydroxymethylation data. Healthy controls (HC) and multiple sclerosis (MS) patients were divided into groups of smokers (S) and non-smokers (NS).

REVISED Supplementary Figure 2

a

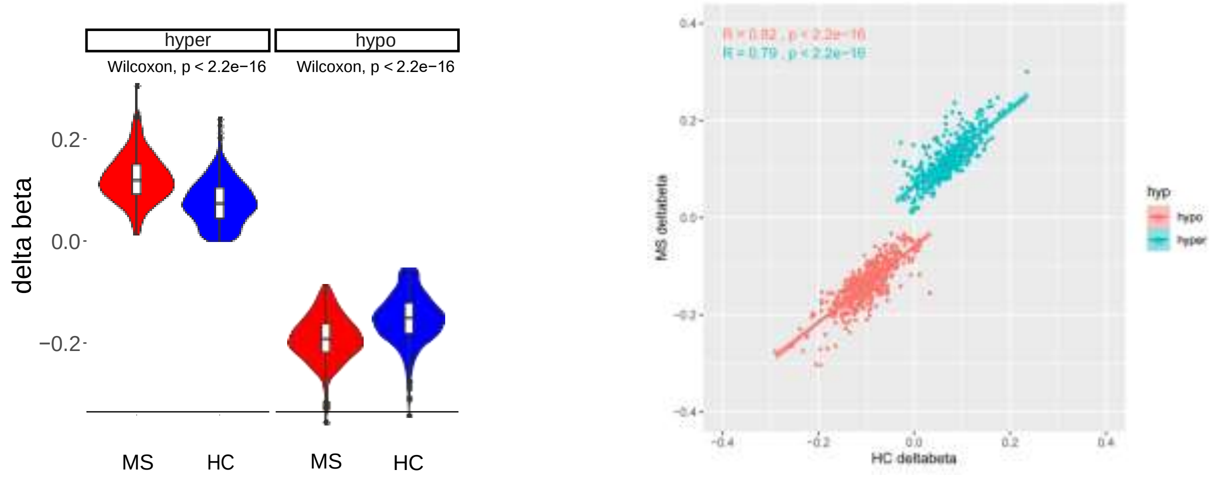

b

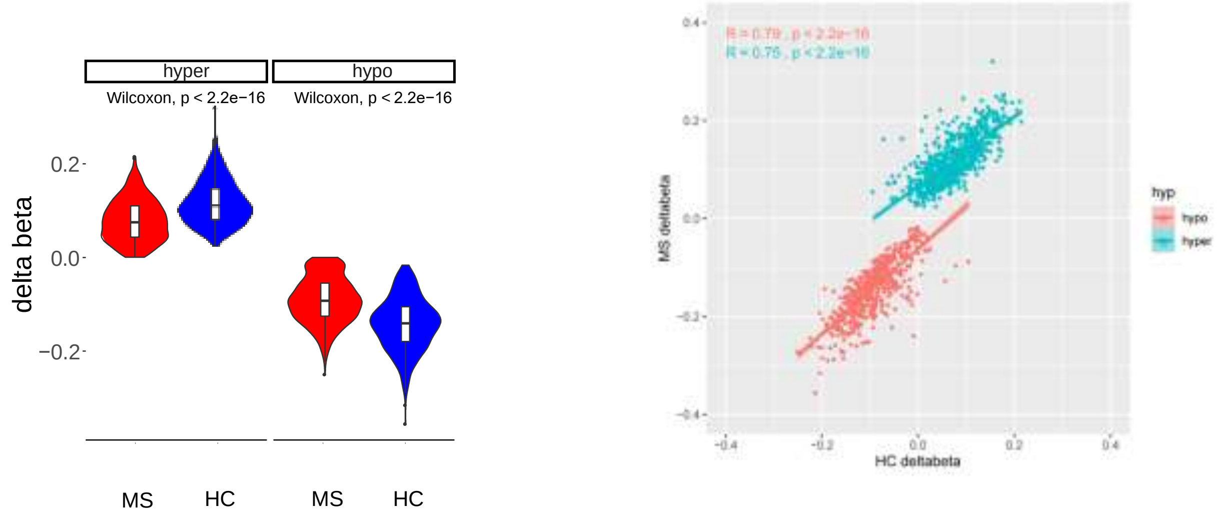

c

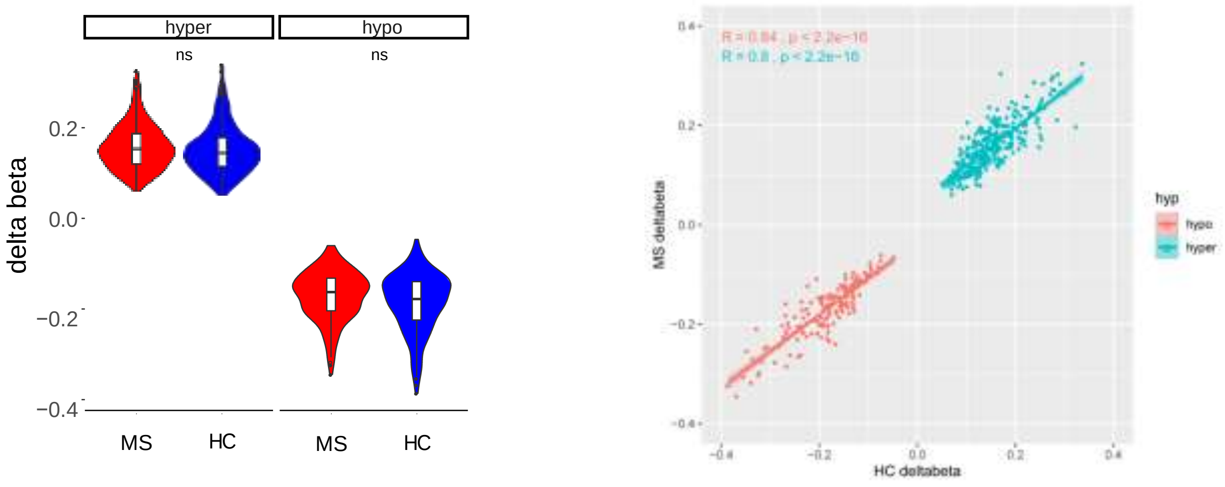

See legend on the next page

Supplementary Figure 2 (end)

d

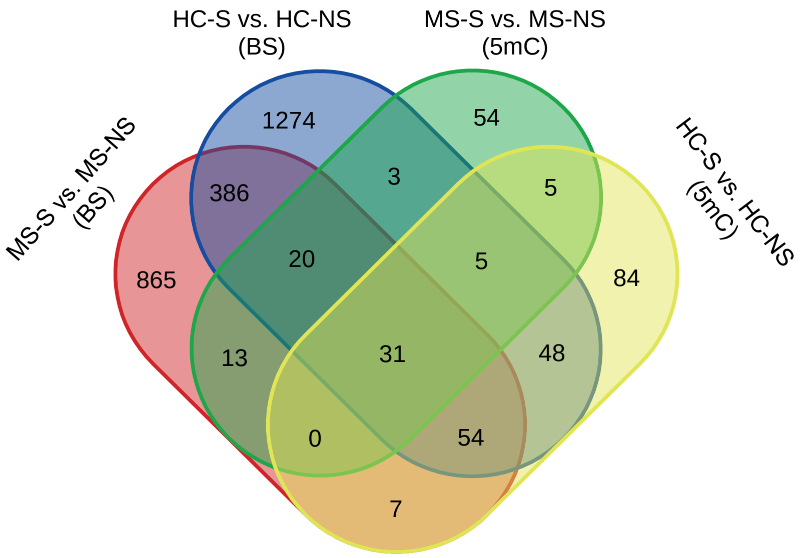

Supplementary Figure 2. Overlap of smoking-associated changes in Multiple Sclerosis (MS) patients and healthy controls (HC).

a-c. Distribution (violin plot, left) and correlation (scattered plot, right) of effect size (Δβ-values) in MS and HC at: (**a**) smoking-associated BS-DMPs in MS patients (MS-S vs MS-NS, adjusted P<0.05) but not significant in HC (HC-S vs. HC-NS, not significant), (**b**) smoking-associated BS-DMPs in HC (HC-S vs. HC-NS, adjusted P<0.05) but not significant in MS (MS-S vs. MS-NS) and (**c**) smoking-associated BS-DMPs in both MS and HC individuals, with delta.beta and beta-values illustrated in the left and right panels, respectively. **d**. Venn diagram of the smoking-associated BS-DMPs and 5mC-DMPs (adjusted p <0.05) in MS patients (MS-S vs-MS-NS) and HC (HC-S vs. HC-NS).

NEW Supplementary Figure 3

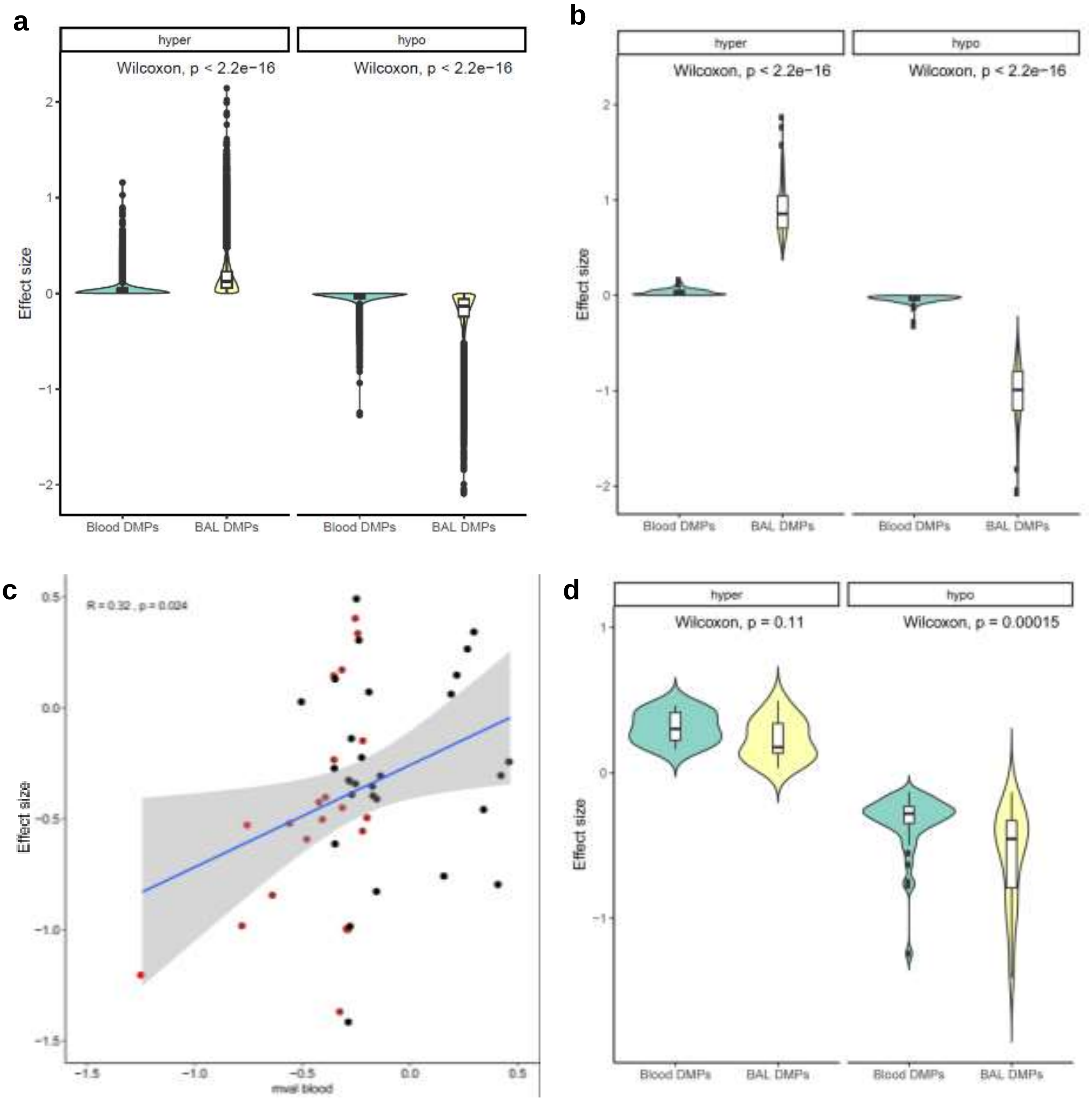

Supplementary Figure 3. Smoking effect in blood DMPs and BAL BS-DMPs of MS individuals.

a. Violin plot showing effect size at probes shared between BAL and blood (n=382277). **b** Violin plot representing all significant BS-DMPs in BAL with the corresponding probes in blood (n=507). **c**. Correlation scatterplot of blood BS- DMPs effect sizes (from M values) at FDR<0.05 (n =50) on the x-axis, and overlapping BAL BS-DMPs values on the y-axis. Red dots represent overlap with significant BAL BS-DMPs at FDR<0.05, while dark red dots represent BAL BS-DMPs at P<0.05. **d**. Violin plot showing effect size at significant BS-DMPs in blood with the corresponding probes in BAL (n=50). Effect size is based on M-values.

Supplementary Figure 4

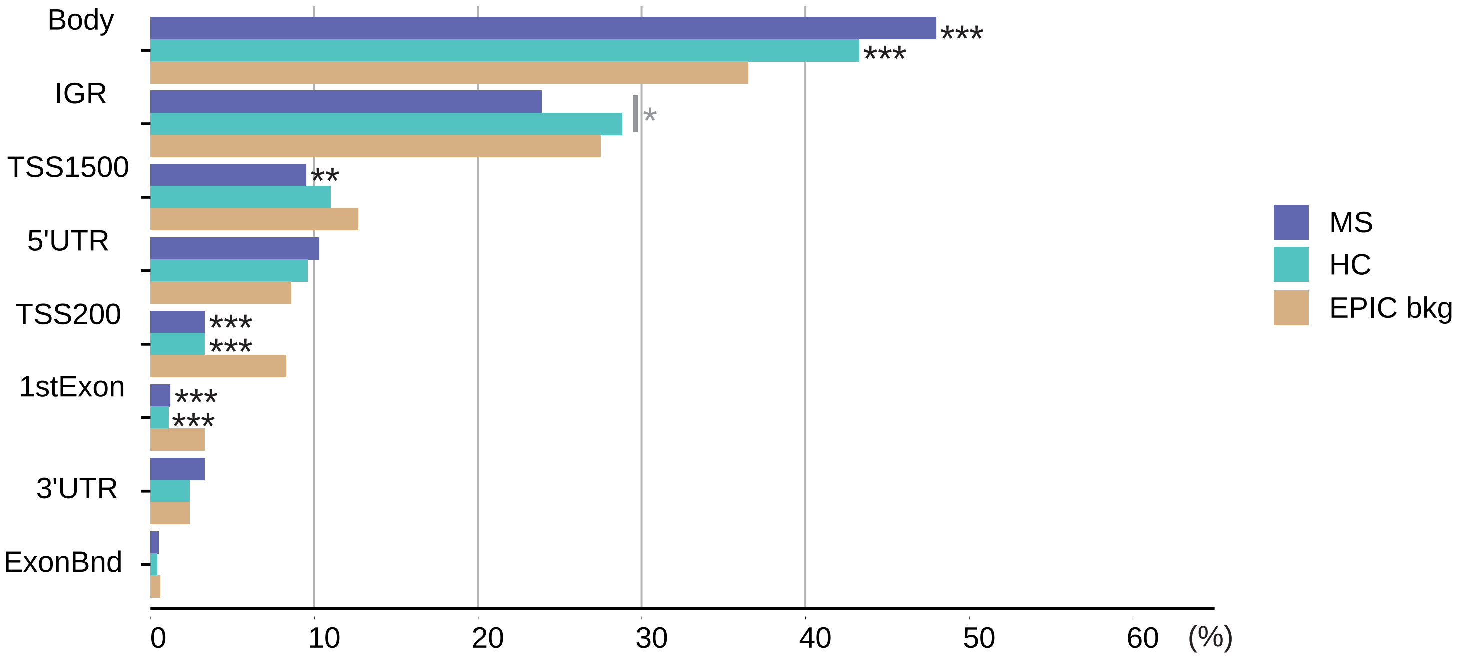

Supplementary Figure 4. Distribution of smoking-associated changes in Multiple Sclerosis (MS) patients and healthy controls (HC) across gene features.

Horizontal barplots representing relative frequencies of BS-DMPs associated with smoking in MS and HC across gene features (TSS1500, TSS200, 1stExon, 5'UTR, Body, 3'UTR, ExonBnd, IGR). Enrichment/depletion analysis was performed using Chi-square test on frequencies, with P-values adjusted for multiple testing (Bonferroni). Asterisk symbols in black (*) represent significance levels from Bonferoni-adjusted Chi-square tests of smoking-associated MS and HC BS-DMP profiles when compared to the EPIC array probe background. Asterisk symbols in grey * represent Bonferoni-adjusted significance levels from smoking-associated BS-DMPs of MS compared to those of HC.
*P-value < 0.05, **P-value < 0.01, ***P-value < 0.001.

Supplementary Figure 5

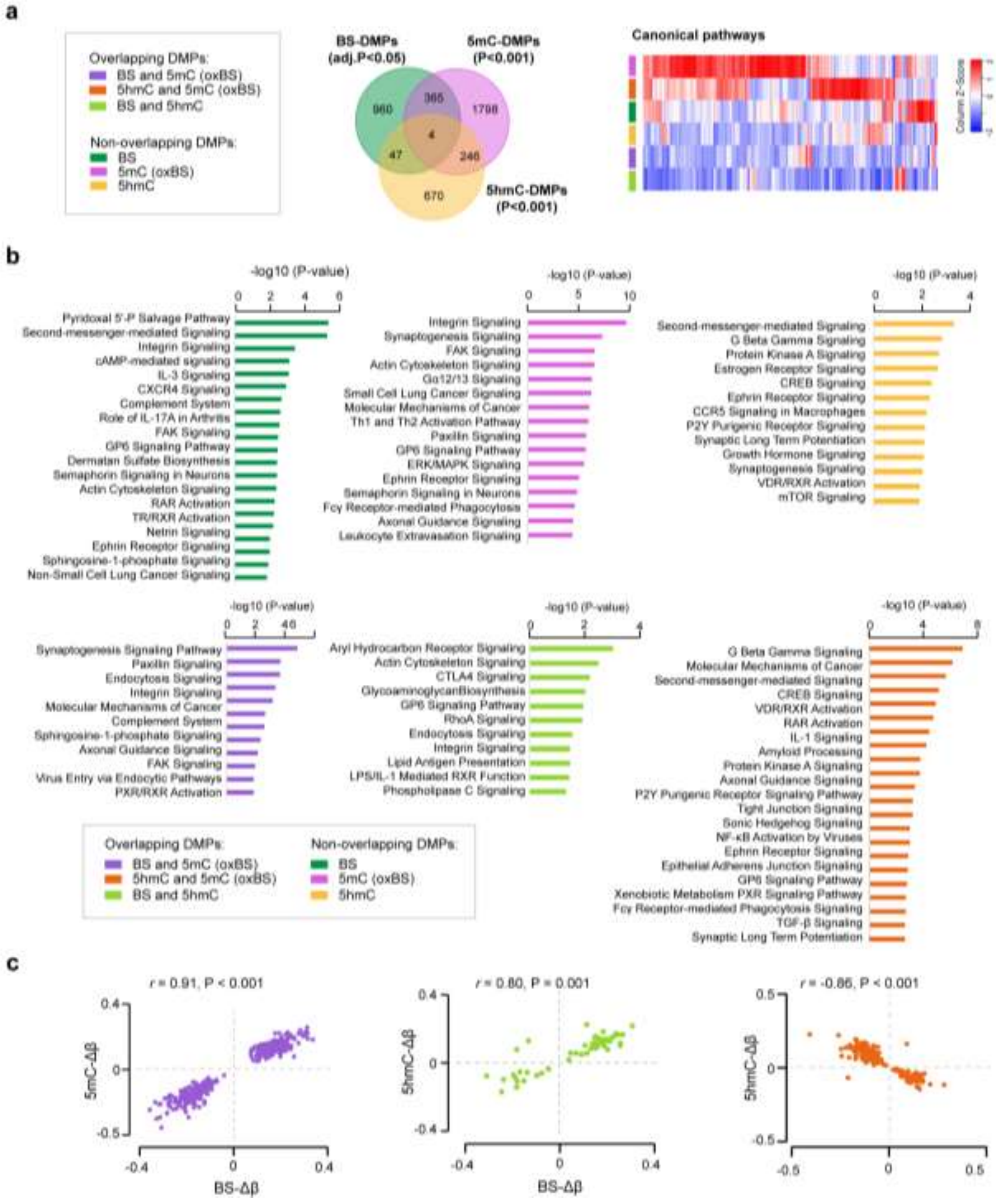

Supplementary Figure 5. Gene ontology (GO) analysis of overlapping and non-overlapping smoking-associated BS, 5mC and 5hmC changes in MS patients.

a. Venn-diagram illustrating the overlap of DMPs between BS (adjusted P<0.05), oxBS (5mC) and 5mC or 5hmC (P<0.001) and heatmap of overlapping Canonical pathways (from Ingenuity Pathway analysis, IPA) with blue to red colors low to high enrichment -log10 (P-value). **b**. Top Canonical Pathways (IPA) enriched in overlapping/non-overlapping DMPs. **c.** Correlation of Δβ-values between overlapping BS-5mC (left panel), BS-5hmC (middle panel) as well as 5mC-5hmC (right panel) DMPs. P-values and Spearman's rank correlation coefficient (*r*) are given.
