# Supplementary material for: Methylome and transcriptome signature of bronchoalveolar cells from multiple sclerosis patients in relation to smoking
Source: Mult Scler. 2020 Jul 30;27(7):1014–26. doi: 10.1177/1352458520943768 (PMC8145441; doi:10.1177/1352458520943768)
Supplement: MSJ943768_supplemental_material – Supplemental material for Methylome and transcriptome signature of bronchoalveolar cells from multiple sclerosis patients in relation to smoking [file MSJ943768_supplemental_material.pdf]

Supplemental Material

Supplementary Method.

Supplementary Table 1. Description of the cohort.

Supplementary Table 2. Smoking-associated (BS, 5mC and 5hmC) differentially methylated CpGs in Multiple Sclerosis (MS) patients and healthy controls (HC).

Supplementary Table 3. Overlapping smoking-associated BS-DMPs in Multiple Sclerosis (MS) between BAL cells and whole blood.

Supplementary Table 4. Gene ontology analyses.

Supplementary Table 5. Differentially expressed genes between Multiple Sclerosis (MS) patients and healthy controls (HC).

Supplementary Figures. Supplementary Figure 1. Analytical workflow. **Supplementary Figure 2.** Overlapping smoking-associated DMPs between Multiple Sclerosis (MS) patients and healthy controls (HC). **Supplementary Figure 3.** Smoking effect in blood and BAL BS-DMPs of MS individuals. **Supplementary Figure 4.** Distribution of smoking-associated changes in Multiple Sclerosis (MS) patients and healthy controls (HC) across gene features. **Supplementary Figure 5.** Gene ontology (GO) analysis of overlapping and non-overlapping smoking-associated BS, 5mC and 5hmC changes in MS patients.
