# Supplementary material for: Methylome and transcriptome signature of bronchoalveolar cells from multiple sclerosis patients in relation to smoking
Source: Mult Scler. 2020 Jul 30;27(7):1014–26. doi: 10.1177/1352458520943768 (PMC8145441; doi:10.1177/1352458520943768)
Supplement: MSJ943768_supplemental_methods – Supplemental material for Methylome and transcriptome signature of bronchoalveolar cells from multiple sclerosis patients in relation to smoking [file MSJ943768_supplemental_methods.pdf]

# Supplementary Methods

## Subjects, bronchoscopy and BAL

Bronchoscopy with accompanying bronchoalveolar lavage (BAL) was performed on 17 MS patients (9 smokers, 8 non-smokers) and 26 healthy control subjects (13 smokers, 13 non-smokers), as previously described. [1] Subjects diagnosed with COPD, asthma, other pulmonary diseases, or other inflammatory conditions were excluded from the study. None of the included subjects had allergy symptoms or airway infections at the time of bronchoscopy. The study was approved by the Regional Ethical Review Board in Stockholm, and all subjects gave their written informed consent. Smokers were defined as current smokers consuming more than 5 cigarettes per day, or with more than 5 pack years (pack year = (cigarettes smoked per day / 20) x years smoking). Non-smokers were defined as having never smoked, or with a smoking cessation more than 5 years ago.

Description of the cohorts used in this study is summarized in Table 1 and details (including smoking, cell proportion and clinical information) are presented in Supplementary Table 1. Briefly, all participants included in this study were females who underwent bronchoscopy with bronchoalveolar lavage, spirometry and clinical assessment, including self-reported smoking information. The majority of MS patients (15/17) were diagnosed with relapsing-remitting MS at the time of sampling, most of them (12/15) being in remission and two patients were diagnosed with progressive MS. At the time of sampling, patients were either not medicated (3/17), under first line IFN-β (8/17) or second line (6/17) treatment (e.g. Natalizumab, Rituximab) at the time of sample collection (Supplementary Table 1). Healthy volunteers represent a subset of a previously described cohort, [2] including only women.

## DNA and RNA extraction

Total RNA and genomic RNA and DNA were extracted from BAL cells using Allprep DNA/RNA/miRNA universal kit from Qiagen following manufacturer's protocol. Concentrations for both DNA and RNA were quantified by Qubit 3 fluorometer. RNA integrity number (RIN) values were obtained using the

RNA 6000 nano chip and the Agilent Bioanalyzer (Agilent Technologies). Samples from MS and controls were processed simultaneously and randomized in downstream analyses.

**DNA methylation sample processing**

To quantify DNA methylation (5mC), DNA hydroxymethylation (5hmC) as well as bulk DNA modifications (total 5mC+5hmC), genomic DNA from BAL cells were processed according to the TrueMethyl kit (Cambridge Epigenetix) workflow, dividing the DNA into two independent reactions, one converted by regular bisulfite treatment (BS) and the other using oxidative bisulfite conversion. Regular bisulfite (BS) treatment allows investigation of total methylation (5mC+5hmC), while oxidative bisulfite (oxBS) treatment permits investigation of true 5mC. Hydroxymethylation values (5hmC) were generated by subtracting normalized oxBS from BS β-values. DNA methylome profiling was carried out using the Infinium HumanMethylationEPIC BeadChip Kit (Illumina), which interrogates over 850000 CpG sites. Methylation arrays were processed by the National Genomics Infrastructure (NGI), Science for Life Laboratory at Uppsala University. MS and healthy control samples were randomized according to smoking-status, age, gender, and cell-proportion and processed together with technical replicates in one run. BS and oxBS samples from each individual were processed together and run on the same array. Technicians performing EPIC arrays were blinded to the MS disease and smoking status during the experiments. The analysts performing statistical analysis were not blinded to disease status and have never altered the diagnosis of samples. Raw intensity IDAT format files were used for subsequent array analysis.

**DNA methylation analysis**

Raw IDAT files were processed using minfi (version 1.28.4)[3, 4] and ChAMP (version 2.12.4)[5] packages in the R environment (software version 3.5.2). Signal intensities were normalized using stratified quantile normalization (SQN), with BS and oxBS data processed separately. Methylation signals were computed as β-values, ranging from 0 (un-methylated) to 1 (fully methylated). BS β-values represent total DNA

methylation (5mC+5hmC), while oxBS β-values only include 5mC. Processing steps are described in Supplementary Figure 1. Probes with a detection P value > 0.01 in > 5% of the samples were excluded, as well as samples with a probe coverage < 95%. Further, Y sex chromosome, SNP-related and cross-reactive probes were removed according to Pidsley et al.[6] As a final preprocessing step, probes and samples that were not shared between BS and oxBS datasets were removed before analyses, resulting in 39 samples with both BS and 5mC methylation values from 734078 probes.

DMP analyses of BS and 5mC signals were performed on M-values (transformed β-values) as recommended by Du et al.[7] We used limma to fit a linear model to each methylation value and empirical Bayes to calculate test statistics for group comparisons of smokers (S) and non-smokers (NS) in multiple sclerosis (MS) patients and healthy controls (HC), adjusting for covariates (age and macrophage fraction). Included covariates were chosen based on their impact on DNA methylation (Table 1). Age was added because of its well-known confounding effect on DNA methylation, and the statistically significant difference observed between MS smokers and HC smokers (Table 1). Alveolar macrophage percentage was included as a measure of BAL composition as alveolar macrophage are the main cell type in BAL, and they were significantly different between groups (Mann-Whitney; p-value < 0.05). Additionally, alveolar macrophage percentage correlates with lymphocyte percentage (Spearman correlation; r = 0.94, p-value < 2.2e-16), and thereby adjusts for both cell type proportions. Similarly, PY was indirectly adjusted for when adjusting for age (Spearman correlation; r = 0.64, p-value = 2.5e-03). Non-significant (neutrophils) and very low percentage (eosinophils and basophils) cell types were excluded, as well as BAL procedural measurements with no impact on DNA methylation (BALF concentration and recovery). Groups were analyzed as follows; in relation to smoking within each group (MS-S *vs.* MS-NS and HC-S *vs.* HC-NS), and between MS patients and healthy controls for both smokers and non-smokers, separately (MS-NS *vs.* HC-NS and HC-S *vs.* MS-S). Probes were considered significantly differentially methylated between groups when the FDR-adjusted (Benjamini-Hochberg) *P*-value < 0.05. Hydroxymethyl (5hmC) methylation values were generated by subtracting normalized 5mC (oxBS) from normalized BS β-values, followed by limma analysis with eBayes. Since oxBS

treatment can introduce negative values, we also performed a second limma analysis where negative values were exchanged for a value close to zero ($1x10^{-7}$). By combining the two methods and only considering overlapping 5hmC DMPs as significant, we aimed to minimize false positives.

CpG locations were divided into the following gene-related categories according to the HumanmethylationEPIC probe annotation (through ChAMP)[5]: TSS1500 (200-1500 nucleotides, nt, upstream of transcription start site, TSS); TSS200 (up to 200 nt upstream of TSS); 5'UTR (5' untranslated region); 1st exon; Body (gene body); ExonBnd (exon boundaries), IGR (intergenic regions), and 3'UTR (3' untranslated region).

Statistical analysis of enriched and depleted differential methylation was performed using Pearson's Chi-squared test on contingency tables of count data. The EPIC array probe background (734078) was used together with significant BS-DMPs, and P-values were adjusted for multiple testing using Bonferroni.

## Generation of RNA sequencing libraries

CDNA libraries were prepared by poly-A capture of 150ng purified total RNA from each individual, using a modified version of SMART-seq2 to adjust for bulk input.[8] Libraries received 6 rounds of pre-amplification before tagmentation using in-house produced TN5 enzyme to yield Illumina Nextera compatible sequencing ready libraries. Libraries were pooled and purified using Ampure XP beads and subsequently sequenced at 125bp paired-end on an Illumina HiSeq 2500.

## Gene expression analysis

RNA sequencing reads were subjected to quality filtering and adapter trimming using in Trim Galore (version 0.6.0) with default parameter settings. The filtered reads were aligned to the transcriptome (Gencode v24) using the pseudoalignment-based Kallisto algorithm (version 0.45.0). For downstream

analysis, only females and groups including a minimum of 7 samples with a RIN value above 7 were included. Genes with >10 normalized read counts were kept. A total of 15 samples from non-smoker individuals passed these criteria, 7 MS and 8 HC, which were used for differential expression analysis using DESeq2 package in R. We adjusted for the covariate age and considered transcripts with nominal *P*-value < 0.05 suitable for GO analysis.

### Gene ontology analyses

Gene ontology (GO) analyses were performed using Ingenuity Pathway Analysis (IPA) (Qiagen) and of the annotated genes harboring 5mC + 5hmC (BS), 5mC (oxBS) and 5hmC changes. Significant BS-DMPs (BH-$P_{adj}$ < 0.05) were used to explore the impact of smoking in MS patients and healthy controls while candidate BS-, 5mC- and 5hmC-DMPs passing unadjusted *P*-value < 0.001 were selected for other instances. Enrichment analyses of differentially expressed genes was performed using transcripts with unadjusted *P*-value < 0.05. IPA was applied using unbiased parameters for all criteria including tissues selection and right-tailed Fisher's exact test was used to calculate *P*-values, with *P* < 0.05 considered statistically significant. We confirmed IPA results using overrepresentation analysis (ORA) (www.webgestalt.org).[9] Visualization of ORA data was performed using REVIGO tool,[10] an algorithm-based method reducing functional redundancies through multidimensional scaling of overrepresented GO terms with semantic similarities in a two-dimensional space, clustering x and y coordinates reflecting closeness of terms. STRING network was generated using STRING database version 10.5 with a minimum level of confidence > 0.4.

## References

1. Olsen HH, Grunewald J, Tornling G, Skold CM and Eklund A. Bronchoalveolar lavage results are independent of season, age, gender and collection site. *PLoS One*. 2012; 7: e43644.

2. Ringh MV, Hagemann-Jensen M, Needhamsen M, et al. Tobacco smoking induces changes in true DNA methylation, hydroxymethylation and gene expression in bronchoalveolar lavage cells. *EBioMedicine*. 2019.
3. Aryee MJ, Jaffe AE, Corrada-Bravo H, et al. Minfi: a flexible and comprehensive Bioconductor package for the analysis of Infinium DNA methylation microarrays. *Bioinformatics*. 2014; 30: 1363-9.
4. Fortin JP, Triche TJ, Jr. and Hansen KD. Preprocessing, normalization and integration of the Illumina HumanMethylationEPIC array with minfi. *Bioinformatics*. 2017; 33: 558-60.
5. Tian Y, Morris TJ, Webster AP, et al. ChAMP: updated methylation analysis pipeline for Illumina BeadChips. *Bioinformatics*. 2017; 33: 3982-4.
6. Pidsley R, Zotenko E, Peters TJ, et al. Critical evaluation of the Illumina MethylationEPIC BeadChip microarray for whole-genome DNA methylation profiling. *Genome Biol*. 2016; 17: 208.
7. Du P, Zhang X, Huang CC, et al. Comparison of Beta-value and M-value methods for quantifying methylation levels by microarray analysis. *BMC Bioinformatics*. 2010; 11: 587.
8. Picelli S, Faridani OR, Bjorklund AK, Winberg G, Sagasser S and Sandberg R. Full-length RNA-seq from single cells using Smart-seq2. *Nat Protoc*. 2014; 9: 171-81.
9. Zhang B, Kirov S and Snoddy J. WebGestalt: an integrated system for exploring gene sets in various biological contexts. *Nucleic Acids Res*. 2005; 33: W741-8.
10. Supek F, Bosnjak M, Skunca N and Smuc T. REVIGO summarizes and visualizes long lists of gene ontology terms. *PLoS One*. 2011; 6: e21800.
